# Ziemssen’s Cyclopœdia, Vol. VI

**Published:** 1877-06

**Authors:** 


*Ziemssen's Cyclopædia of the Practice of Medicine.* Vol. VI. Diseases of the Circulatory System, with Diseases of the Lips, Cavity of the Mouth, and Soft Palate, and Whooping Cough. By ALBERT H. BUCK, M. D., American Editor. New York: Wm. Wood & Co.

This volume contains a thousand pages, of which four hundred and seventy-eight are devoted to diseases of the heart and pericardium. Prof. Samuel S. Rosenstein, of the University of Leyden, contributes the portion which treats of the endocardial and valvular diseases of the heart, and it shows a carefulness and completeness which is very creditable. He recognizes three forms of endocardial inflammation; acute diphtheritic or ulcerative, sub-acute verrucose, and chronic contracting or schleotic endocarditis. The last form is generally associated in advanced life with atheromatous disease of the arterial system and the process of its development he considers is not to be distinguished from endarteritis, except in the contracting and indurating effects which follow endocardial inflammation: it is of insidious development and has the same etiology with atheroma, *e. g.*, old age, gout, alcoholism.

Diphtheritic or ulcerative endocarditis is of a typhoid or pyæmic character, and gives rise to the multiple capillary form of embolism. Sub-acute verrucose endocarditis is the more frequent form following articular rheumatism, and the emboli which result are of larger size and produce more noticeable local effects. Very frequently the development of valvular lesions is throughout of a chronic nature, and the primary causation not to be determined. The various secondary lesions which arise in different organs from embolism and congestion are described. Valvular lesions, their symptoms, diagnosis and prognosis are carefully and clearly presented.

The maximum intensity of an aortic regurgitant murmur is located at the third right sterno-costal articulation, instead at the fourth left sterno-costal articulation and diffused downwards to the apex, as given by Prof. Flint. In mitral regurgitation, the murmur is said to be propagated to the left auricle, and is heard in its auricular appendix which overlaps the pulmonic orifice; the transmission of the sound to the left of the apex laterally, and beneath the scapula behind, according to Flint, is not mentioned. In other respects, he is in accord with our own observers. The acuteness of American clinical observations and diagnosis of disease of the heart and lungs is a very just ground for national pride. We are pleased to notice Prof. Rosenstein advocates a quite discreet use of digitalis, of iron acetate, potassium acetate, valerian, hydrocyanic acid and other like remedies. Prof. Lebert, of Vevay, treats of Congenital diseases of the Heart. Prof. Schroetter, of Vienna, treats of Changes of Position of the heart and disease of its substance, and does not accept to the degree that Prof. Quincke, of Berne, who presents the Diseases of the Arteries, Veins, and Lymphatics, does, the idea ably supported by Seitz, and various authors, that severe physical exertion may give rise to endocardial or endarterial inflammation, which may be followed by fatal valvular disease. Dr. Bauer, of Munich, treats very fully of Pericarditis, as does also Dr. Steffen, of Stettin, upon Whooping Cough. Prof. Vogel of Dorpat, and author of a work on Diseases of Children, a translation of which was published in this country a few years ago, treats of Diseases of the Lips and Cavity of the Mouth, giving particular attention to Parotitis. Prof. Wagner, of Leipsic, author of the recent work on General Pathology, completes the volume with Diseases of the Soft Palate, among which the most noticeable and interesting, is perhaps the portion which treats of the various forms of croupous and diphtheritic pharyngitis. The completeness of the descriptions of disease in its various forms and with its complications and sequelæ, which is given in Ziemssen's volumes has been hitherto unattained, and make the series a most desirable acquisition.

